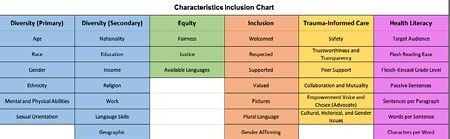# 756 Key Characteristics and Best Practices for Burn Patient Education: A Modified Scoping Review and Comparison

**DOI:** 10.1093/jbcr/irae036.298

**Published:** 2024-04-17

**Authors:** Grace E Smith

**Affiliations:** St. Catherine University, Eagan, MN

## Abstract

**Introduction:**

Within the Western industrialized countries, the incidence of burns is roughly 40,000 per year in the United States. There are currently 69 verified burn centers in the United States. Providing optimal burn care requires centers to provide an intensive team, increased knowledge, and various equipment.1 Because of the high incidence of burn injuries in the United States, educational material is provided to patients during their recovery. However, 36% of the United States adult population has limited health literacy skills, likely, many patients don't understand all of the written materials they receive. Individuals with a lower health literacy are more likely to be readmitted to the hospital after discharge. Adapting patient education materials to meet personalized characteristics can increase patient’s recovery.

Adapting patient education materials may address diversity, equity, inclusion (DEI), trauma-informed care (TIC), and health literacy.

**Methods:**

A modified scoping review and PRISMA guidelines5 were used to answer two questions: 1. What are the key characteristics and best practices in DEI, TIC, and Health Literacy in patient education for established burn centers? 2. What are the characteristics of patient education in established burn centers in DEI, TIC, and Health Literacy?

**Results:**

A Key Characteristic Chart was developed, and a sample of best practice recommendations for mental health, gender neutral, language, and grade level based on the literature. After, two collections were reviewed using the Key Characteristic Chart. A sample of DEI, TIC, and Health literacy characteristics provides data on two collections.

**Conclusions:**

This comparison of the two collections revealed multiple characteristics relating to DEI, TIC, and health literacy. Continuation of developing adapted patient education from the two collections may improve patient-centered care relating to DEI, TIC, and health literacy.

**Applicability of Research to Practice:**

Providing patient-specific rather than general information can increase knowledge and satisfaction and decrease anxiety.6 Healthcare professionals need in-service training on providing patient education through multiple teaching strategies. There should also be a focus on minimizing trauma to burn survivors from the earliest point of contact and throughout the recovery journey. This includes providing patient education that shows characteristics of TIC to emphasize safety from physical and emotional harm and re-traumatization.